# Polyethylene spinout in the Attune® Cruciate-Retaining Rotating-Platform (CR RP) total knee arthroplasty performed with a cruciate-sacrificing and measured-resection technique

**DOI:** 10.1186/s43019-020-00057-0

**Published:** 2020-07-22

**Authors:** Cillian J. Keogh, David Mulcahy, Declan Reidy, David E. Beverland, James A. Harty

**Affiliations:** 1 Department of Trauma and Orthopaedic Surgery, Cork University Hospital/South Infirmary Victoria University Hospital, Wilton, Cork, T12 DC4A Republic of Ireland; 2 Department of Orthopaedic Surgery, Bon Secours Hospital Cork, College Road, Cork, Republic of Ireland; 3 Musgrave Park Hospital, Stockman’s Lane, Belfast, BT9 7JB Northern Ireland, UK

**Keywords:** Rotating, Platform, Cruciate, Retaining, Gap, Balancing, Attune®, LCS®, Polyethylene, Spinout

## Abstract

**Introduction:**

Polyethylene (PE) spinout is a known but uncommon complication when using a mobile-bearing (MB) total knee arthroplasty (TKA) design. Sacrificing the posterior cruciate ligament (PCL) is within the manufacturer’s recommendations for the Attune® Cruciate-Retaining Rotating-Platform (CR RP) knee design.

**Aim:**

To discuss the potential aetiology and prevention of spinout in the Attune® CR RP knee.

**Methods:**

We used a retrospective radiological review from two centres reporting a higher rate of spinout in the Attune® CR RP knee using a cruciate-sacrificing and measured-resection technique when compared to a gap-balancing technique. Three hundred and thirty-two patients were evaluated over a 3-year period.

**Results:**

There were 8 out of 279 (2.86%) cases of spinout in our first cohort of patients using a measured-resection technique. There were 0 out of 53 cases of spinout in our second cohort of patients where a gap-balancing technique was used. One spinout was reduced closed, the other seven were initially revised to a thicker RP insert of the same design. Of these seven, three underwent a further revision TKA and one patient required a knee fusion/arthrodesis.

**Conclusions:**

This study reports a higher incidence of PE spinout in the Attune® CR RP TKA when a measured-resection technique in combination with PCL resection is performed. We recommend a gap-balancing technique with conservative soft-tissue release if the surgeon is planning to sacrifice the PCL in the Attune® CR RP.

## Introduction

Primary total knee arthroplasty (TKA) includes fixed-bearing (FB) and mobile-bearing (MB) designs. In the FB design the polyethylene (PE) insert is fixed to the tibial component while, in the MB design, the PE insert can rotate on the tibial baseplate. This was initially designed to address the problems of wear due to high contact stress on PE inserts in FB knee designs [1]. The most widely used MB has been the Low Contact Stress (LCS®) rotating platform (RP) (DePuy Synthes, Chester, PA, USA). This has an excellent clinical track record and reported outcomes [2]. It continues to be the model for MB knee systems.

The Attune® (DePuy Synthes, Chester, PA, USA) knee system was introduced to address the unmet needs in TKA of 10–20% patient dissatisfaction rates [3, 4]. The design includes the Attune® Gradius™ curve with a gradually reducing femoral radius. The aim of this curve is to create a smooth transition during knee-bending and produces high stability of the knee by minimising unnatural sliding of the femur on the tibia. This should aid stair ascent and descent. The initial data supporting the Attune® knee system is very positive. Attune® has a Kaplan-Meier estimate of cumulative percentage probability of first revision at 3 years of 1.52% according to the National Joint Registry (NJR). This is comparable to the LCS® Complete (1.67%) (DePuy Synthes, Chester, PA, USA), the Triathlon® (1.53%) (Stryker, Kalamazoo, MI, USA) and the NexGen® (1.43%) (Zimmer Biomet, Warsaw, IN, USA) [5].

The manufacturer of the Attune® knee system allows surgeons to sacrifice both the anterior cruciate ligament (ACL) and the posterior cruciate ligament (PCL) with the Attune® Cruciate-Retaining Rotating-Platform (CR RP) configuration as it is based around the model of LCS®. The surgical technique guide for Attune® states that the CR RP configurations can be used for either a cruciate-retaining (CR) or a cruciate-sacrificing (CS) application [6].

A recognised complication which is unique to RP devices is the potential for spinout of the PE insert from beneath the femoral implant. The biggest series with the LCS® [7] reported 26 spinouts using the LCS® RP over a 24-year period in 8373 cases, giving an incidence of 0.31%. In this series, in all of the cases both cruciate ligaments were sacrificed and a gap-balancing, as opposed to a measured-resection, technique was used [8]. In the first 8 years of this series the incidence of spinout was 0.58%; during this period the authors routinely released collateral ligaments in both varus and valgus knees to achieve soft-tissue balance in extension. In the last 16 years they stopped releasing the collateral ligaments [9, 10] and the incidence fell to 0.2% (12 out 5994).

The term spinout is used because either the medial or lateral side of the PE insert remains in joint. This is in contrast to a dislocation where neither would be in joint, as can occur with a fixed-bearing TKA. This means that the bearing moves or ‘spins out’ on the side which is most slack, which is more often laterally.

## Aims

The aim of this study was to consider the aetiology, prevention and possible risk factors for spinout in the Attune® CR RP.

## Methods

Data was collected retrospectively from three orthopaedic surgeons who performed the Attune® CR RP primary TKAs in two separate orthopaedic centres. The inclusion criterion was any patient who received an Attune® CR RP primary TKA in either centre between November 2015 and May 2018. Data was collected retrospectively from the patient’s clinical notes to identify age, sex, date of surgery, surgical technique, time to spinout, and method of treatment. Anteroposterior (AP) and lateral X-rays of the knee were reviewed at the time of spinout to assess the direction of spinout.

In the first cohort of patients, a measured-resection technique in combination with PCL resection was performed. For our second cohort of patients a gap-balancing technique in combination with PCL resection was performed.

Surgery was performed using a medial parapatellar approach to the knee joint. In all cases the ACL and PCL were resected. The patella was never resurfaced.

**Fig. 1:**
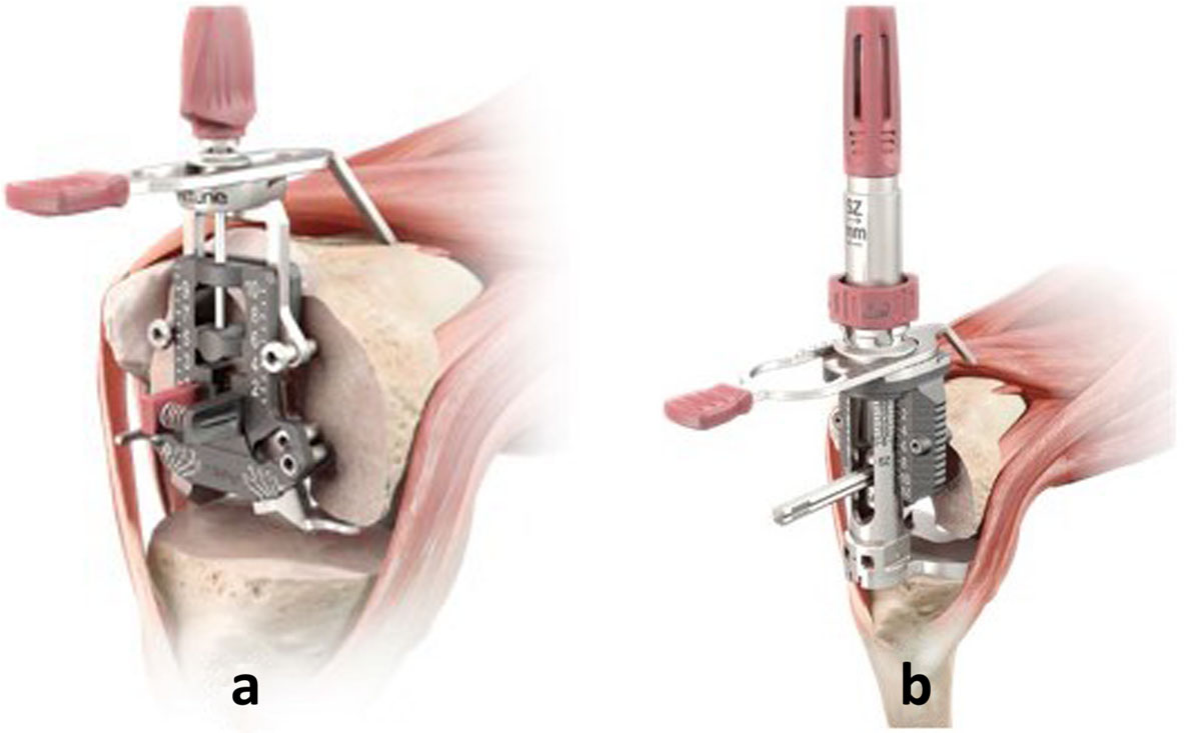
**a** Shows the Attune® measured sizer instrument for a measured resection surgical technique. **b** Shows the Attune® balanced sizer instrument used for gap-balancing surgical technique

For our first cohort of patients a tibia-first approach was used for the bony cuts using an extramedullary jig held with pins proximally referencing the ACL footprint and centred midway between the malleoli distally. This was followed by femoral cuts using measured-resection technique landmarks (Whiteside’s lines) to determine femoral component rotation. The goal for the tibial-implant position was 90° to the AP tibial axis with 5–7° of posterior tibial slope. Soft-tissue release for deformity was conservative. The collateral ligaments and popliteus tendon were not released irrespective of deformity, instead opting for a posterolateral/posteromedial capsulotomy. Both surgeons had initially attempted to use the balance sizer as shown but had found it difficult to use and, thus, converted to a measured-resection technique intra-operatively (Fig. 1a).

For our second cohort of patients a tibia-first approach was also used for the bony cuts using an extramedullary jig and again referencing the ACL footprint and centred midway between the malleoli distally. The distal femoral cut was then performed using an intramedullary jig which was set to give an initial distal femoral cut of 5° with respect to the anatomic axis. The extension gap was then assessed using a spacer block. The balance sizer as shown in Fig. 1b was then used to size the femoral component and set its rotation based on ligament tension.

Postoperatively, patients received a tailored physiotherapy rehabilitation regimen which included full weight-bearing mobilisation and range-of-motion exercises as early as day 0 postoperatively. After discharge from hospital, patients attended the surgeon’s outpatient department at 6 weeks postoperatively and an arthroplasty nurse-led clinic at 1 year, 2 years and 5 years postoperatively. Patient-related outcome measures (PROMS) were also recorded at these postoperative appointments.

## Results

The overall incidence of spinout in the first cohort of patients using a measured-resection technique was 8 per 279 cases or 2.86%. In our second cohort of patients using a gap-balancing technique there were 0 spinouts in 53 cases.

The indication for TKA in all eight cases of spinout was primary osteoarthritis. Data for each patient is summarised in Table 1. There were two male patients while six (75%) patients were female. The mean age at time of TKA for the spinout patients was 69.6 years (range 53–84 years).

**Table 1:** Shows summarised data on all patients who sustained a spinout

Case no.	Age	Sex	BMI	Time to spinout (days)	Femoral component size	Pre-operative deformity	Dislocation direction	Outcome
1	63	Female	41	29	5	5° varus	Posteromedial	Hinged prosthesis
2	83	Female	28.4	14	6	11° valgus	Posterolateral	Hinged prosthesis
3	67	Female	34	2	5	Neutral	Posterolateral	Polyethylene exchange
4	64	Female	45	2	4	8° varus	Posterolateral	Knee fusion
5	84	Female	33	43	4	19° valgus	Posterolateral	Polyethylene exchange
6	53	Male	19.5	19	5	11° valgus	Posteromedial	Polyethylene exchange
7	71	Male	26.4	134	5	7° valgus	Posterolateral	Polyethylene exchange
8	72	Female	29.5	29	3	5° valgus	Posteromedial	VVC revision prosthesis

*BMI* Body Mass Index, *VVC* varus-valgus constrained

Of the eight spinout cases, five patients (62.5%) had a pre-operative valgus deformity (mean 10.6°; range 5–19°), two patients (25%) had a varus deformity (mean 6.5°, range 5–8°) and one patient had a neutral knee alignment.

Spinout was diagnosed at a mean of 34 days (1 month) following TKA (range 2–134 days). Six cases were diagnosed within the first month. The direction of spinout was posterolateral in five patients and posteromedial in three patients, none were anterior.

**Fig. 2:**
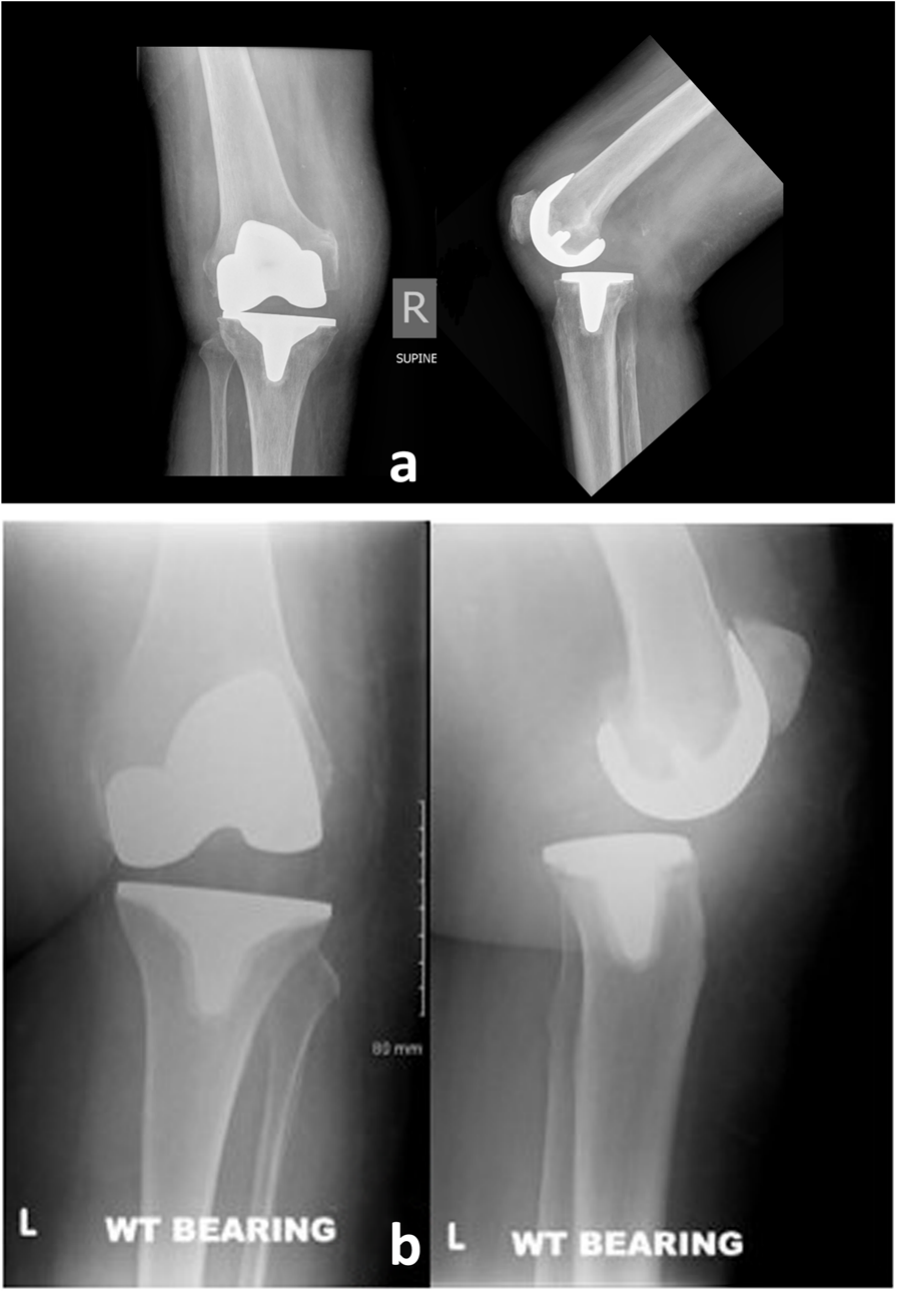
**a** X-ray showing an example of a posterolateral polyethylene spinout; there is posterior translation of the tibia on the lateral X-ray and on the anteroposterior (AP) view the lateral side of the joint space is closed down. **b** X-ray showing an example of a posteromedial polyethylene spinout; there is also posterior translation of the tibia on the lateral X-ray but on the AP view the medial side of the joint space is closed down

A posterolateral spinout is shown in Fig. 2a; there is posterior translation of the tibia on the lateral X-ray and on the AP view the lateral side of the joint space is narrowed. Fig. 2b demonstrates a posteromedial spinout.

In one of the patients the dislocated PE insert could be relocated by closed means and was treated in a knee immobiliser for 8 weeks. The other seven were initially revised to a thicker RP insert of the same design. Of these seven, three underwent a further revision to a varus-valgus constrained TKA and one patient required a knee fusion/arthrodesis.

PROMS for all 279 patients in the first cohort were recorded using the Knee Injury and Osteoarthritis Outcome Score (KOOS). The mean KOOS for all patients was 81.9 at the 1-year follow-up and 85.5 at the 2-year follow-up.

Other documented complications of the 279 TKAs in our first cohort of patients were; one case of infection postoperatively needing washout and PE liner exchange, and two cases of pulmonary embolism needing anticoagulation.

## Discussion

The Attune® CR RP is very similar in design to the LCS® rotating platform. However, as the former name suggests, the surgeon has the option to retain the PCL when using the Attune® design whereas the PCL is routinely released in the LCS®. Also, the LCS® technique has very much a gap-balancing philosophy, whereas with the Attune® system the surgeon has the option of either a measured-resection technique or a ligament/gap-balancing technique.

Historically, for all TKAs, posterior stability or resisting posterior translation of the tibia relative to the femur has been a key design aim. This fell broadly into two types; namely, PCL retention (CR) and posterior stabilised (PS). In the former the PCL resists posterior translation and in the latter the cam and post, which substitute for the PCL, resist posterior translation.

Predictably, as with all TKAs, both mobile- and fixed-bearing [11] dislocation or spinout is most often posterior. Theoretically, PCL retention in a MB design should reduce the risk of posterior-bearing spinout. This is due to the considerable posterior translation distance required by the tibia to allow the femur to ride over the anterior lip of the PE insert. The other advantage of PCL retention is that it adds to the collateral stability of the knee, particularly if either collateral ligament is released. Consequently, in an LCS® knee with a sacrificed PCL and a released collateral ligament there can be significant flexion-gap laxity, which, in some cases, can overwhelm the deep dish resulting in spinout.

LCS® depends on its ‘deep dish’ configuration to provide posterior stability when the PCL is released. The posterior translation of the tibia relative to the femur is resisted by the anterior lip of the PE insert. In the Attune® knee system the jump height of the anterior lip of the PE insert is lower than the LCS®, but for most sizes the difference is less than 1 mm. This design change was to facilitate femoral rollback and deep flexion when the PCL is intact. It would, therefore, appear that the Attune® is less resistant than the LCS® to posterior translation and spinout. However, an internal DePuy analysis of force required to sublux the femoral condyle over the anterior lip (posterior translation) of the insert showed that the Attune® actually required 3% more force to sublux when compared to LCS®. This increased anterior stability is achieved via a smaller sagittal radius of the femoral component and insert, which produces a more curved surface to resist femoral-component subluxation. This will, however, only work if the flexion gap is balanced and well-tensioned. If the flexion gap is loose, then the extra force required becomes irrelevant and the dominant mechanical factor becomes anterior jump height.

The other factor to appreciate with respect to the mechanism of spinout is that it is very unlikely to occur in extension. For example, in the LCS® for the first 30° of flexion the PE insert and the femoral component are fully congruent. In both the Attune® and the LCS® designs, the change in the radius of curvature of the femoral component means that the PE insert becomes increasingly less congruent with flexion. This then increases intrinsic AP instability and, thus, spinout occurs as a result of flexion-gap instability.

As we know, the primary aetiology of spinout is flexion-gap instability with the commonest patient factor being a pre-operative valgus knee [11]. In the normal knee, in flexion, the lateral compartment is looser than the medial. In the valgus knee this looseness can be accentuated by release of the popliteus or lateral collateral ligament. The impact of the release of these two structures is increased if the PCL is also released.

Malrotation of the femoral component is a potent cause of instability and dissatisfaction. Proper rotation of the femoral component is essential to obtaining a balanced flexion gap [12]. Reliability on anatomical landmarks for measured resection is not always possible and can lead to malrotation of the femoral component [8, 13–15]. Due to its independence from obscured or poorly identified anatomical landmarks, gap-balancing may offer superior reliability [16]. Harvey et al. have demonstrated that relying on measured resection alone in the Attune® TKA may result in femoral component malrotation [17]. They found that a gap-balancing technique using the tensioner device gave more reliable external rotation. From a technical perspective when using the gap-balance sizer, it is critical that the distal femoral cut and tibial cuts are kept flush with the device at 90° of flexion during flexion-gap balancing.

One of the key observations from this series of eight spinouts was their postoperative management. In only one case did it prove possible to achieve stability with a closed reduction and immobilisation. The other seven cases had an initial revision to a thicker insert but, of these, three went on to undergo a revision to a constrained implant and one underwent a knee arthrodesis.

In contrast, the LCS® experience reported by Diamond et al. [7] showed that in their second cohort of 5994 patients, where gap-balancing without release of collateral ligaments was performed, there were 12 cases of spinout (0.2%). First of all, the incidence was 14 times less than in this series but also 11 of the 12 cases were treated successfully with closed reduction and immobilisation with only one patient going on to a full revision.

In our second cohort of patients, similarly to Diamond et al., all of the cases were performed using a gap-balancing technique in combination with PCL resection; however, the collateral ligaments were never released. This led to no documented spinouts in our second cohort of patients and a spinout rate of only 0.2% in the cohort followed by Diamond et al. [7].

Interestingly, a NJR implant summary report published in August 2018 showed only one spinout in a total of 19,818 Attune® TKA cases performed over a 6-year period [18]. The philosophy of soft-tissue balancing in England, Wales, Northern Ireland and the Isle of Man leans towards a gap-balancing technique.

In contrast, in our first cohort of patients a measured-resection technique was used in combination with CS, which, in 2.86% of cases, led to spinout as a result of flexion-gap instability. Interestingly both surgeons using this technique were previous LCS® users, each with over 15 years of experience using a tibial-first gap-balancing technique with a less than 1% incidence of spinout with that implant.

## Conclusion

This study reports a higher incidence of PE spinout in the Attune® CR RP TKA when the measured-resection technique in combination with PCL resection is performed. During a measured-resection procedure, if the PCL has to be sacrificed then the surgeon should consider a fixed bearing if they have any concerns about flexion-gap stability. In keeping with the manufacturer’s recommendations, it is also perfectly acceptable to routinely sacrifice the PCL (CS) with the Attune® CR RP but, if so doing, then we recommend using a gap-balancing technique. It is our further recommendation that with a gap-balancing technique a conservative soft-tissue release should be performed.

## Data Availability

Available on request.

## References

[1] Malinzak RA, Small SR, Rogge RD, Archer DB, Oja JW, Berend ME (2014). The effect of rotating platform TKA on strain distribution and torque transmission on the proximal tibia. J Arthroplast.

[2] Hopley CD, Crossett LS, Chen AF (2013). Long-term clinical outcomes and survivorship after total knee arthroplasty using a rotating platform knee prosthesis: a meta-analysis. J Arthroplast.

[3] Baker PN, Rushton S, Jameson SS, Reed M, Gregg P, Deehan DJ (2013). Patient satisfaction with total knee replacement cannot be predicted from pre-operative variables alone: a cohort study from the National Joint Registry for England and Wales. Bone Joint J.

[4] Bourne RB, Chesworth B, Davis A, Mahomed N, Charron K (2010). Comparing patient outcomes after THA and TKA: is there a difference?. Clin Orthop Relat Res.

[5] National Joint Registry for England, Wales and Northern Ireland. 15th Annual Report, 2018. http://www.njrreports.org.uk/Portals/0/PDFdownloads/NJR%2015th%20Annual%20Report%202018.pdf. Accessed on 28 Nov 2018

[6] Attune® knee system surgical technique guide. http://synthes.vo.llnwd.net/o16/LLNWMB8/US%20Mobile/Synthes%20North%20America/Product%20Support%20Materials/Technique%20Guides/DSUSJRC03161437%20Rev%205_ATTUNE%20Cemented%20Primary%20Knee%20Surgical%20Tech.pdf

[7] Diamond OJ, Doran E, Beverland DE (2018). Spinout/dislocation in mobile-bearing total knee arthroplasty: a report of 26 cases. J Arthroplast.

[8] Daines BK, Dennis DA (2014). Gap balancing vs. measured resection technique in total knee arthroplasty. Clin Orthop Surg.

[9] Pagoti R, Beverland D (2017) Soft tissue balancing without ligament release in complex total knee replacement. Thieme Medical and Scientific Publishers Pvt, Belfast, Ltd ISBN: 978–93–86293-09-1

[10] Pagoti R, O’Brien S, Doran E, Beverland D (2017). Unconstrained total knee arthroplasty in significant valgus deformity: a modified surgical technique to balance the knee and avoid instability. Knee Surg Sports Traumatol Arthrosc.

[11] Lebel B, Lewallen D (2010) Total knee arthroplasty dislocation: incidence, etiology, and management. J Bone Jt Surg Br 92-B(Suppl II)

[12] Yoshiya S, Matsui N, Komistek RD, Dennis DA, Mahfouz M, Kurosaka M (2005). In vivo kinematic comparison of posterior cruciate-retaining and posterior stabilized total knee arthroplasties under passive and weight-bearing conditions. J Arthroplast.

[13] Dennis DA, Komistek RD, Kim RH, Sharma A (2010). Gap balancing versus measured resection technique for total knee arthroplasty. CORR.

[14] Katz MA, Beck TD, Silber JS, Seldes RM, Lotke PA (2001). Determining femoral rotational alignment in total knee arthroplasty: reliability of techniques. J Arthroplasty.

[15] Kim CW, Lee CR, Gwak HC, Kim JH, Kwon YU, Kim DY (2020). The effects of surgical technique in total knee arthroplasty for Varus osteoarthritic knee on the rotational alignment of femoral component: gap balancing technique versus measured resection technique. J Knee Surg.

[16] Huang T, Long Y, George D, Wang W (2017). Meta-analysis of gap balancing versus measured resection techniques in total knee arthroplasty. Bone Joint J.

[17] Harvey RA, Hossain M (2017). Femoral component rotation in the Attune TKR, balanced technique and measured resection instrumentation.

[18] ATTUNE® CR Knee and ATTUNE® PS Knee NJR Implant Summary Report August 2018. http://synthes.vo.llnwd.net/o16/LLNWMB8/US Mobile/Synthes North America/Product Support Materials/PDF-PowerPoints/JOINT RECONSTRUCTION/092206–180822 ATTUNE® Knee Implant Summary Report Presentation Cert.pdf

